# The Acute-Phase Proteins Serum Amyloid A and C Reactive Protein in Transudates and Exudates

**DOI:** 10.1155/MI/2006/47297

**Published:** 2006-02-08

**Authors:** Alessandra M. Okino, Cristiani Bürger, Jefferson R. Cardoso, Edson L. Lavado, Paulo A. Lotufo, Ana Campa

**Affiliations:** ^1^Departamento de Patologia, Análises Clínicas e Toxicológicas, Centro de Ciências da Saúde, Universidade Estadual de Londrina, CEP 86051-990 Paraná, Brazil; ^2^Departamento de Análises Clínicas e Toxicológicas, Faculdade de Ciências Farmacêuticas, Universidade de São Paulo, CEP 05508-900 São Paulo, Brazil; ^3^Núcleo de Investigações Químico-Farmacêuticas, Centro de Ciências da Saúde, Universidade do Vale do Itajaí, CEP 88302-202 Santa Catarina, Brazil; ^4^Departamento de Fisioterapia, Centro de Ciências da Saúde, Universidade Estadual de Londrina, CEP 86051-990 Paraná, Brazil; ^5^Hospital Universitário, Universidade de São Paulo, CEP 05508-900 São Paulo, Brazil

## Abstract

The distinction between exudates and transudates is very important in the patient management. Here we evaluate whether the acute-phase protein serum amyloid A (SAA), in comparison with C reactive protein (CRP) and total protein (TP), can be useful in this discrimination. CRP, SAA, and TP were determined in 36 exudate samples (27 pleural and 9 ascitic) and in 12 transudates (9 pleural and 3 ascitic). CRP, SAA, and TP were measured. SAA present in the exudate corresponded to 10% of the amount found in serum, that is, the exudate/serum ratio (E/S) was 0.10 ± 0.13. For comparison, the exudate/serum ratio for CRP and TP was 0.39 ± 0.37 and 0.68 ± 0.15, respectively. There was a strong positive correlation between serum and exudate SAA concentration (*r* = 0.764;*p* < 0.0001). The concentration of SAA in transudates was low and did not overlap with that found in exudates (0.02-0.21 versus 0.8–360.5 g/mL). SAA in pleural and ascitic exudates results mainly from leakage of the serum protein via the inflamed membrane. A comparison of the E/S ratio of SAA and CRP points SAA as a very good marker in discriminating between exudates and transudates.

## INTRODUCTION

Serum amyloid A (SAA) and C-reactive protein (CRP) are acute-phase proteins predominantly produced and secreted by hepatocytes [1]. Other cells including lymphocytes, monocytes, and macrophages can also produce these proteins [2]. The induction of SAA and CRP synthesis is triggered by a number of cytokines, chiefly IL-6, which is released from a variety of cell types, but mainly from macrophages and monocytes at inflammatory sites [3].

Although several studies have investigated the serum levels of the acute-phase proteins CRP and SAA in diseases [4–8], few have focused on the levels of these type of proteins in effusions, that is, transudates and exudates. Serum CRP is widely used as a marker of inflammation and tissue injury [9, 10]. Although CRP is also found in exudates and it has been proposed for differentiating diseases, the diagnostic application of this finding has not been fully explored. For SAA, information regarding its presence and possible diagnostic use in effusions is not available at all.

Effusions are commonly classified as transudate or exudate according the Light's criteria that is based in determinations of total protein and lactate dehydrogenase [11]. However, as the distinction between exudates and transudates are very important in the patient management, there is a continuous interest in the evaluation of other biochemical parameters [12–16]. Plasma ligands of SAA and CRP are distinct (for review see [17, 18]), supporting that they can differently leakage through membranes. The purpose of this study was to evaluate the presence of SAA in pleural and ascitic fluids, compare it with CRP, and evaluate the possibility of using these acute-phase proteins to discriminate effusions.

## METHODS

SAA, PCR, and total protein (TP) were determined in pleural and ascitic effusions and corresponding serum samples taken from adult patients hospitalized in the Hospital Universitário/Universidade Estadual de Londrina (Paraná, Brazil) and Hospital Universitário, Universidade de São Paulo (São Paulo, Brazil). Pleural and ascitic effusions were classified as transudates or exudates according to the Light's criteria; effusion to serum total protein ratio > 0.5, an effusion lactate dehydrogenase (LDH) value > 200 U/L, or a fluid to serum LDH ratio > 0.6 [11]. All of the patients gave their informed consent to participate in the study, which was approved by the Ethics Committee of the Hospital Universitário of Universidade Estadual de Londrina (CEP 111/01) and the Ethics Committee of the Faculdade de Ciências Farmacêuticas of Universidade de São Paulo (Ofício CEP no 64).

Serum and exudate samples were taken from 36 hospitalized patients, 27 with pleural effusions and 9 with ascitic effusions. The pleural exudates were caused by pneumonia (18 cases), tuberculosis (7 cases), and neoplasia (2 cases). The ascitic exudates resulted from peritoneal tuberculosis (3 cases), peritonitis (1 case), neoplasia (2 cases), cirrhosis (2 cases), and chronic hepatitis (1 case). Serum and transudate samples were taken from 12 patients, 9 with pleural effusions and 3 with ascitic effusions. The pleural transudates were caused by congestive cardiac failure (5 cases), hepatic cirrhosis (2 cases), and renal failure (2 cases). The ascitic transudates resulted from hepatic cirrhosis (2 cases) and undefined ascite (1 case).

The samples were centrifuged at 3000 rpm for 10 minutes and stored at −70°C for up to 10 months. CRP was measured by immunonephelometry, using a Beringher Nephelometer 100 Analyzer and a Dade Behring kit (Marburg, Germany). SAA was measured by ELISA, using a Tridelta Phase kit (Maynooth, Co, Kildare). Total protein was measured by a modified biuret method in an automated Dimension Clinical Chemistry System analyzer, using the Dade Behring kit.

The Statistical Package for the Social Sciences (SPSS version 9.0) program was used to carry out a distribution analysis by the Kolmogorov-Smirnov test, while the correlation coefficients were determined according to Spearman's rank-correlation test and by multiple-level regression analysis. A *p* value of < 0.05 was considered significant.

## RESULTS

Because the comparative analysis showed no differences in the serum and exudate concentrations of SAA, CRP, and TP with respect to the origin and location of the effusion, a collective descriptive analysis for SAA, CRP, TP, and their effusion/serum ratio was made for exudates (Table 1) and for transudates (Table 2). The concentrations of serum and effusion SAA and CRP varied over a broad interval, especially in exudates.

Likewise, the effusion/serum ratio for CRP and SAA also varied over a broad interval, specially in exudates (Table 1) when compared with transudates (Table 2). The exudate displayed, on average, 10% and 39% of SAA and CRP present in the serum, respectively.

**Figure 1:**
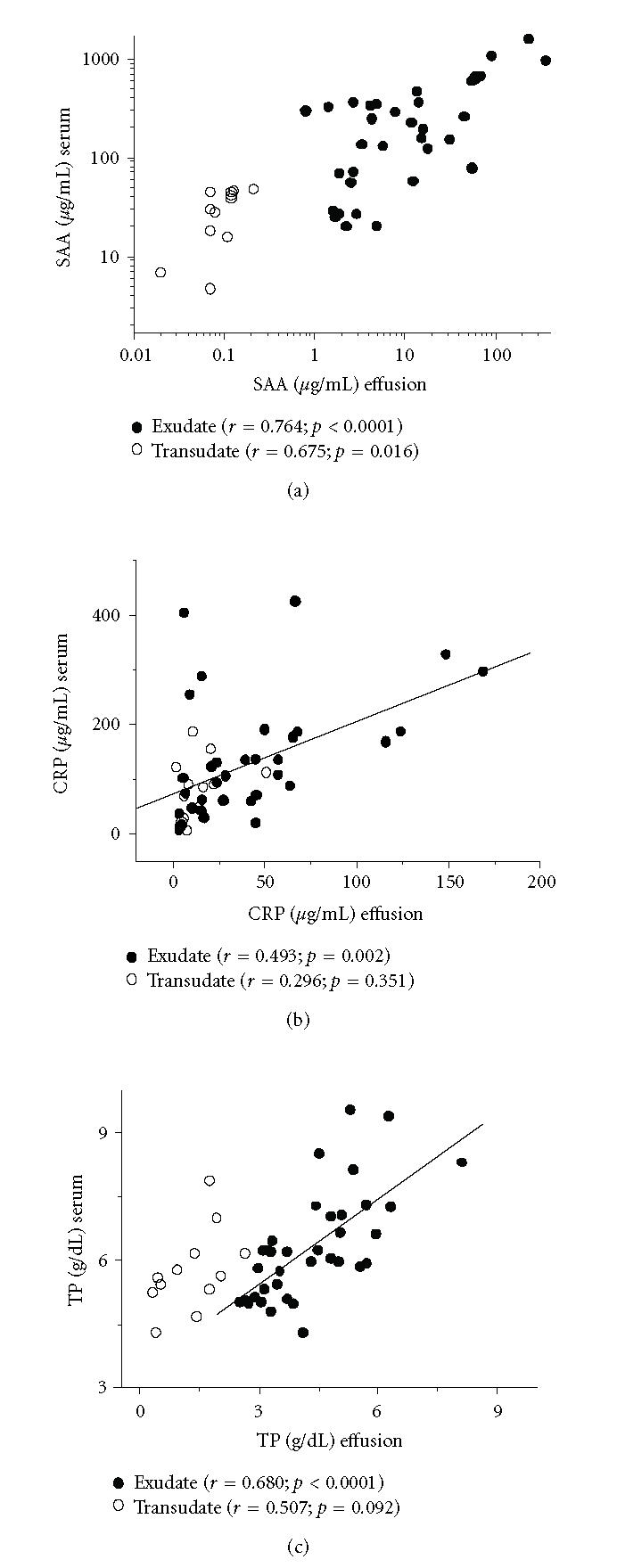
Correlation between effusion and serum for SAA (a), CRP (b), and TP (c). Exudates and transudates were from 36 and 12 patients, respectively. When shown the line represents the regression for exudates.

**Figure 2:**
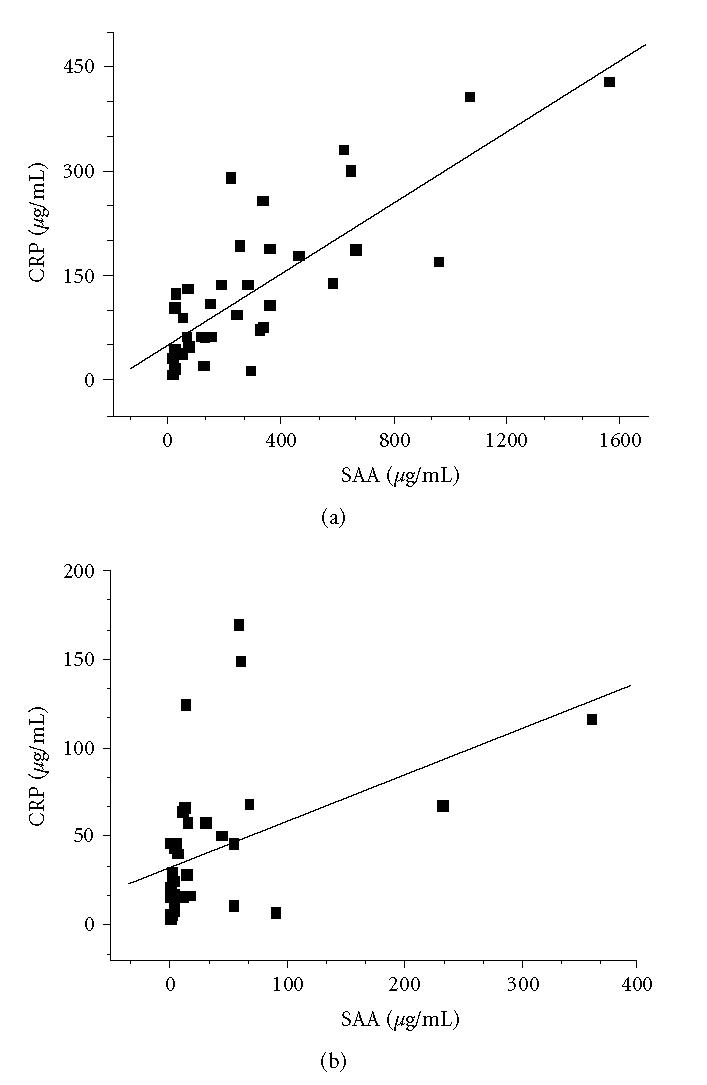
Correlation between CRP and SAA for (a) serum (*r* = 0.796; *p* < 0.0001) and (b) exudate (*r* = 0.449; *p* = 0.007) in 36 patients.

The correlation analysis of serum and effusion for SAA, CRP, and TP (Figure 1) using Spearman's test showed, in exudates, a stronger correlation for SAA than for CRP and TP (note the log scale for SAA). Although SAA and CRP were highly correlated in serum, they were only slightly correlated in exudate (compare Figures 2(a) and 2(b)).

**Figure 3:**
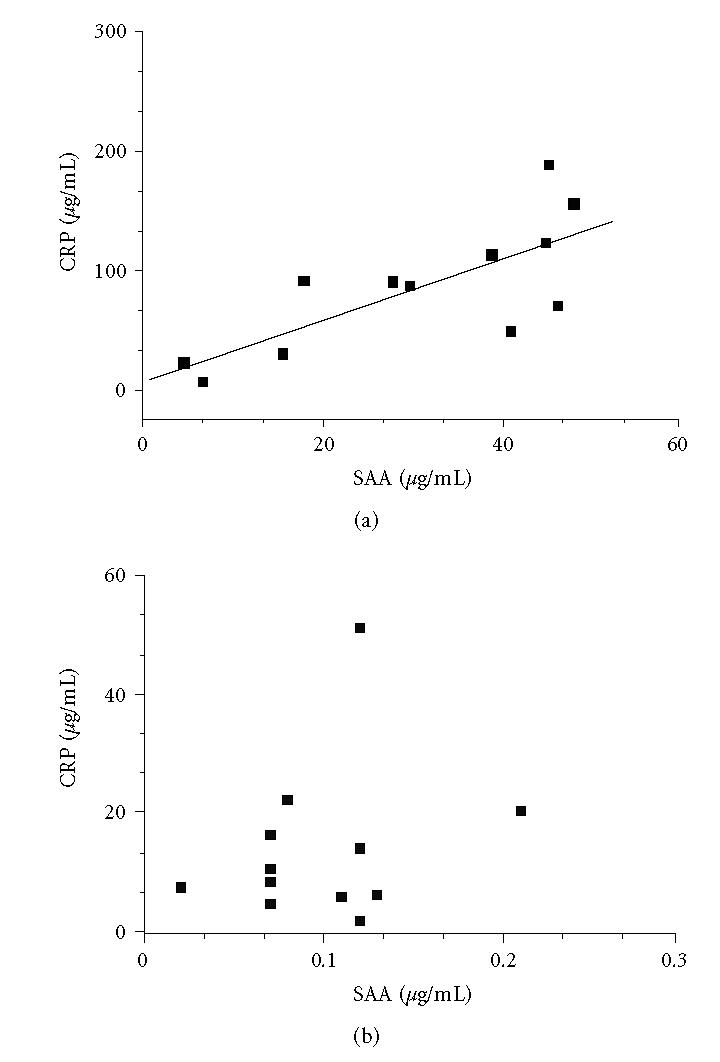
Correlation between CRP and SAA for (a) serum (*r* = 0.749; *p* = 0.005) and (b) transudate (*r* = 0.247; *p* = 0.438) in 12 patients.

Albeit the concentration of SAA in transudates was low, it was possible to identify a correlation between serum and transudate (see the value of *r* in Figure 1(a)). Curiously this correlation was observed only for SAA and was not present for CRP. There was a good correlation between CRP and SAA in serum but not in transudates (Figure 3). The CRP/SAA ratio varied over a broad interval in both exudates and transudates (Table 3).

**Figure 4:**
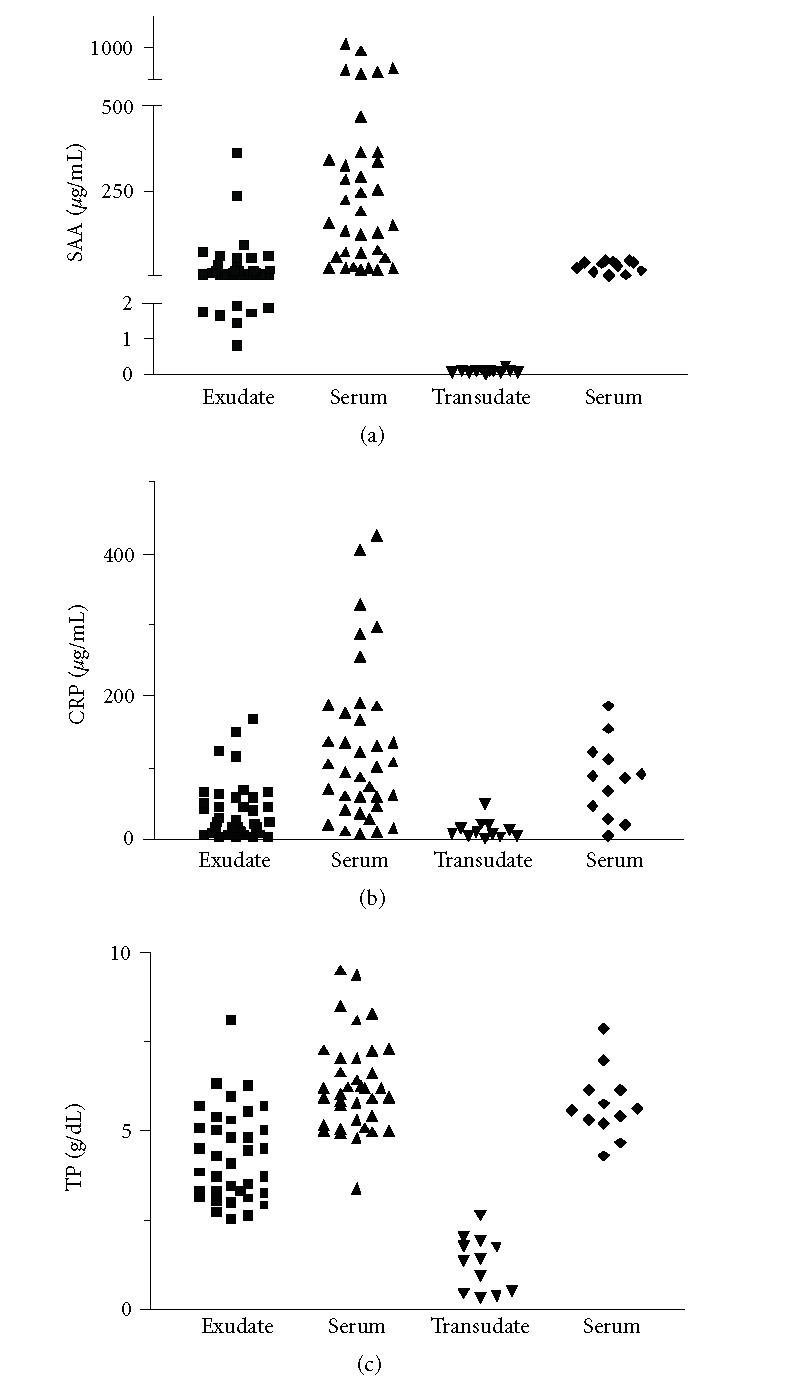
Individual values of (a) SAA , (b) CRP, and (c) TP in serum and effusions (36 exudates and 12 transudates).

The analysis of the individual values of SAA, CRP and TP found in exudates, transudates, and respective serum showed that the simple measurement of SAA in the effusion was able to discriminate transudate from exudate (Figure 4). This did not occur with the other parameters.

## DISCUSSION

**Figure 5:**
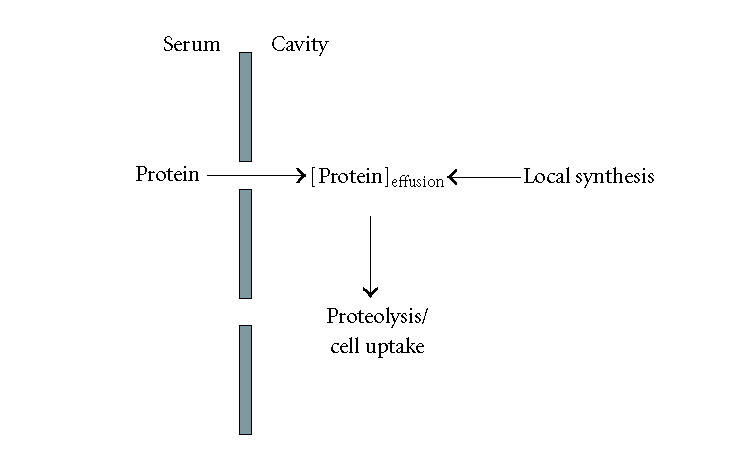
Factors affecting the concentration of a given protein in an exudate.

The concentration of a given plasma protein in an effusion will depend on its leakage through the pleural and peritoneal membranes, and for some of them, on local synthesis. Alternatively, proteolysis and cell uptake will contribute to a decrease in protein concentration (Figure 5). The synthesis of SAA in the inflammatory focus is expected. Indeed, activated monocytes express and release SAA [19]. Thus, although local synthesis of SAA can not be excluded, the positive correlation between serum and effusion (Figure 1(a)) indicates a strong contribution of serum to the pool of SAA present in the effusion. The proteolysis of SAA in the exudate and the association of SAA with cells is expected because: (i) fragments of SAA are present in synovial fluid of arthritic patients [20], (ii) SAA undergoes proteolysis by lysosomic enzymes of neutrophils [21], and (iii) there is a specific receptor for SAA in macrophages [22] and neutrophils [23]. These same processes occur with CRP [24, 25]. Based on the correlations found for CRP and SAA in serum and in exudate (Figure 2), we assume that these proteins cross the membrane and/or are fragmented to varying degrees, that is, the passage of CRP through membranes occurs more readily than the passage of SAA, or the proteolysis or uptake of SAA by cells is greater than that of CRP.

It is important to note that the concentration of SAA in exudates is only 10% of that present in serum. However, this concentration is sufficiently high to trigger the biological effects described for this protein, for instance, 1 μg/mL of SAA is sufficient to trigger the mRNA expression and the release of TNF-α and IL-8 from human neutrophil cultures [26–28]. Besides the induction of cytokine synthesis and release [26–29], SAA primes neutrophils [30] and is involved in cell migration [31]. CRP binds to phosphocholine and thus recognize foreign pathogens [32]. Ligand-bound CRP also activates the complement pathway [33]. The presence of CRP and SAA in exudates supports a key role of these proteins in the activation of immune responses and/or in the repair of host tissues.

The broad range of concentrations for SAA and CRP in exudates observed in this study was expected and probably reflected different phases of the inflammatory disease. Even though, this study suggests the potential value of SAA in the characterization of exudates, as already proposed for CRP [9, 34]. Although several molecular markers have been proposed for the discrimination of exudates and transudates [35, 36], a definitive marker has not yet been found. SAA determinations are relatively simple, rapid, and inexpensive and in this study we find that SAA can undoubtedly contribute to the discrimination of an exudate.

## Figures and Tables

**Table 1:** Descriptive analysis of parameters determined in serum and exudate samples and their exudate/serum ratio (E/S) (*n* = 36).

Parameters	Serum		Exudate		E/S	
	Range	Mean ± SD	Range	Mean ± SD	Range	Mean ± SD

SAA (μg/mL)	20.0–1567.1	307.1 ± 341.9	0.8–360.5	33.9 ± 70.5	0.003–0.710	0.10 ± 0.13
CRP (μg/mL)	7.3–426.1	130.0 ± 109.4	3.0–168.6	40.4 ± 41.6	0.015–2.210	0.39 ± 0.37
TP (g/dL)	4.3–9.5	6.3 ± 1.3	2.5–8.1	4.3 ± 1.3	0.50–0.98	0.68 ± 0.15

**Table 2:** Descriptive analysis of parameters determined in serum and transudate samples and their transudate/serum ratio (T/S) (*n* = 12).

Parameters	Serum		Transudate		T/S	
	Range	Mean ± SD	Range	Mean ± SD	Range	Mean ± SD

SAA (μg/mL)	4.7–47.9	30.5 ± 15.9	0.02–0.21	0.1 ± 0.04	0.002–0.01	0.004 ± 0.003
CRP (μg/mL)	5.4–187.0	84.5 ± 54.5	1.5–50.8	13.9 ± 13.2	0.01–1.36	0.28 ± 0.36
TP (g/dL)	4.3–7.9	5.7 ± 0.9	0.3–2.6	1.3 ± 0.8	0.08–0.42	0.22 ± 0.12

**Table 3:** Descriptive values for the CRP/SAA ratio in exudates (*n* = 36) and transudates (*n* = 12) and respective serum.

	CRP/SAA	
	Range	Mean ± SD

Exudate	0.1–31.6	4.7 ± 5.8
Serum	0.1–4.2	0.8 ± 0.9

Transudate	12.3–423.3	162.6 ± 132.7
Serum	0.8–5.0	2.8 ± 1.3
